# Multiple Diversity of Mitochondrial Cytochrome *b* Amino Acid Sequences of the Same Length in Animals

**DOI:** 10.3389/fmolb.2020.00102

**Published:** 2020-06-17

**Authors:** Alexander A. Zamyatnin, Tatiana A. Belozerskaya

**Affiliations:** Bach Institute of Biochemistry, Federal Research Centre “Fundamentals of Biotechnology”, Russian Academy of Sciences, Moscow, Russia

**Keywords:** protein, cytochrome *b*, primary structure, amino acid residue number, UniProt database, Swiss-Prot database, TrEMBL database

## Abstract

The size of natural peptide molecules (proteins) can be considered as the number of amino acid residues *p* (protein length). The aim of the work was to analyze the region of existence and occurrence of natural amino acid residue sequences formed as a result of matrix synthesis on the *p* scale. The object of the study was the Swiss-Prot database consisting of more than 5.6 × 10^5^ primary peptide structures, which were fully determined (complete sequence). Sequences containing non-standard amino acid residues, as well as identical copies of sequences, were removed from them. The remaining 463,450 different sequences with a length of 2–35,213 residues were used for further analysis. It was shown that the protein lengths of different biological domains and kingdoms are characterized by different regions of existence, and the profile shapes of the obtained curves are close to a number of known distributions. At the same time, they have sharp high peaks, indicating the existence of a large number of specific molecules with the same protein length. One of these peaks characterizes more than 1,000 different sequences of mitochondrial cytochrome *b* molecules at *p* = 379. Such examples may indicate that the most perfect protein lengths were selected in the evolutionary process to perform this function. As a result, many protein molecules with different sequences of the same length and characterized by the same functions were formed.

## Introduction

In the general case, peptides are called natural substances consisting of submolecular blocks (20 standard amino acid residues) connected by a peptide bond (Sewald and Jakubke, 2002). Obviously, the minimal linear peptide is a chemical structure in which two amino acid residues are connected by one peptide bond (dipeptide). Natural peptide sequences (primary structures) containing many amino acid residues (polypeptides) are commonly called proteins, and long molecules of this type can consist of tens of thousands of amino acid residues. Thus, the size (length) of a molecule of peptide nature can vary over a wide-ranging number of amino acid residues.

There is a great variety of amino acid residue sequences as well. This is based on a broad spectrum of possible combinations of 20 standard amino acid residues. With an increase in the length of the peptide sequence *p*, the number of such combinations of *N*_*p*_ rapidly grows in accordance with the well-known formula

$$\begin{array}{l} N_{p}=\;20^{p}, \end{array}$$

(where *p* is a number of amino acid residues in one molecule). In the case of dipeptides, *p* = 2, *N*_2_ = 400, tripeptides *p* = 3, *N*_3_ = 8,000, tetrapeptides *p* = 4, *N*_4_ = 160,000, etc., and at the end of the interval 2 ≤ *p* ≤ 50, characterizing oligopeptides (Zamyatnin, 1991a, 2018a), i.e., at *p* = 50, this value reaches the value *N*_50_ = ~10^34^. Of course, not all combinations of amino acid residues can exist in nature, but still the variety of possible primary structures should be accepted as gigantic. In this work, we studied the distribution of the number of different (unique) natural peptide sequences along the length, i.e., by the number of amino acid residues *p*.

## Materials and Methods

To obtain information on natural peptide amino acid residue sequences, the UniProt database is most often used, which combines manually annotated and reviewed data (Swiss-Prot database Bairoch and Boeckmann, 1991 and the TrEMBL database Kneale and Kennard, 1984 on primary structures obtained as a result of the translation (Tr) of nucleotide sequences into the language of amino acids (automatically annotated and not reviewed).

The ability to perform different procedures and analyses is provided on the UniProt database website. The UniProt database program tools were used in our work to highlight all and specific amino acid residue sequences (option: Search), exclusion of sequence fragments (option: Sequence > Fragment > Sequence complete), extraction of specific sequences with a given number of amino acid residues *p* (option: Sequence > Sequence length > from *p*_1_ to *p*_2_), exclusion of identical sequences (option: Protein page > Similar proteins > 100% identity), and sorting protein names and sequences by different characteristics (standard UniProt table sorting).

The procedures described in this work were performed on a high-speed server, which allows you to process large amounts of information. The main part of the study described below was carried out on the Swiss-Prot database data.

## Results

### Protein Length Distribution in UniProt Database

**Figure 1:**
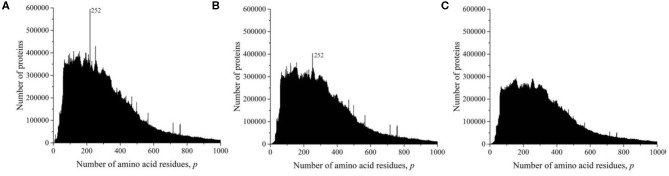
Dependence of the number of sequences of the UniProt database on the number of amino acid residues. (**A**) All sequences, **(B)** sequences that are not fragments, and **(C)** sequences that are not fragments, do not contain nonstandard (O and U) or unidentified (X) amino acid residues, and without identical full copies.

At the time of the study, the UniProt database contained information on 159,022,877 amino acid residue sequences obtained for species of archaea, prokaryotes, and eukaryotes. The minimum number of amino acid residues *p* = 2 contains three oligopeptides of a different origin, and the maximum (*p* = 74,488) contains one translated bacterial polypeptide. Despite such a large range of *p*, most sequences are concentrated in the range 2 ≤ *p* ≤ 1,000 (154,685,385, i.e., more than 97%). The distribution in this interval is shown in Figure 1A. A characteristic feature of this distribution is several sharp peaks, indicating a significantly larger number of sequences with a given number of amino acid residues *p*, compared with neighboring values of *p*, e.g., a peak is especially distinguished at *p* = 252. An examination of primary structures corresponding to this peak represents a large set of very functionally different proteins.

However, this distribution does not give information on the true number of occurrences of amino acid residue sequences in nature. These data are incorrect, since the UniProt database contains, in addition to complete sequences, data on 15,495,873 incomplete sequences (almost 10%) that are fragments. The distribution shown in Figure 1B characterizes an array of 143,527,004 natural peptide amino acid residue sequences after excluding all fragments from consideration. Several peaks are also detected in this distribution, but their values and position are different from those shown in Figure 1A.

As it transpired during the study, the UniProt database also includes sequences containing amino acid residues indicated by letters that are not used to describe standard residues: O (hydroxyproline), U (α-aminobutyric acid), and X (unidentified). Sequences that contain these letters were also excluded from consideration.

The UniProt database also contains a large number of completely identical sequences, obtained, as a rule, as representatives of different, but taxonomically close living organisms. In order for the analysis to be limited to considering only different sequences, all duplicates were also excluded from consideration (Figure 1C). It can be seen that the peak at *p* = 252 is absent in Figure 1C because there are many duplicates in all sequences of UniProt database (Figures 1A,B). This peak contains more than 57,000 sequences of viral matrix proteins 1, but many of them are the same, e.g., the protein sequence (V9SYV1) of influenza A virus [A/Peru/PER334/2011(H3N2)] is found in more than 10,000 entries of UniProt database.

### Protein Length Distribution in Swiss-Prot Database

**Figure 2:**
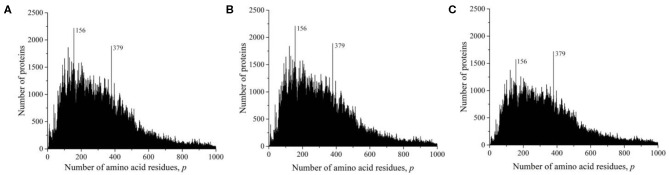
Dependence of the number of sequences from the Swiss-Prot database on the number of amino acid residues. **(A)** All sequences, **(B)** sequences that are not fragments, and **(C)** sequences that are not fragments, do not contain nonstandard (O and U) or unidentified (X) amino acid residues, or without identical full copies.

At the time of the study, the Swiss-Prot database contained information on 560,118 amino acid residue sequences obtained as representatives of archaea, prokaryotes, and eukaryotes. The minimum number of amino acid residues *p* = 2 contained two oligopeptides of a different origin, and the maximum (*p* = 35,213) contained the mouse protein titin (Church et al., 2009). It transpired that, despite such a large range of *p*, most sequences are concentrated in the range 2 ≤ *p* ≤ 1,000 (542,302, i.e., ~97%). The distribution of all sequences in this interval is shown in Figure 2A. A characteristic feature of this distribution is the many sharp peaks indicating a significantly larger number of sequences with a given number of amino acid residues *p*, compared with neighboring *p*-values. Among them, two peaks are especially distinguished at *p* = 156 and 379. An examination of the protein corresponding to these peaks showed that, at *p* = 156, 2,216, sequences represent a large set of very different proteins. However, in the case *p* = 379, out of 1,889 proteins, 1,048 (two-thirds) are mitochondrial cytochrome *b*. This value is approximately equal to that part of the peak that rises above the total mass of protein lengths in Figure 2A.

As in the case of the UniProt database, we deleted all incomplete sequences that are fragments in the Swiss-Prot database. The distribution of complete sequences is shown in Figure 2B. This figure does not differ much from Figure 2A, since fragmented sequences from the Swiss-Prot database make up only 1.6% (9,167) of the total number of data in this database. Some difference is noticeable only in the field of oligopeptides, i.e., at small values of *p*.

We obtained a significantly changed distribution after removing sequences with non-standard and unidentified amino acid residues, and also after removing duplicates (Figure 2C). Note that the peak value at *p* = 156 decreased significantly, while at *p* = 379, it remained unchanged. Detailed data on the number of amino acid residue sequences taken for the already described and subsequent analyses are collected in Table 1.

**Table 1:** Data on the number of amino acid residue sequences in various taxonomic groups and species on the Swiss-Prot database.

**Taxon groups, species (common names)**	**Taxon groups, species (Latin names)**	**All on the Swiss-Prot database**	**Without fragments**	**Without duplicates**	***p*_**min**_**	***p*_**max**_**
All		560,118	550,951	463,450	2	35,213
Eukaryotes	*Eukaryota*	189,697	182,592	174,063	2	35,213
Animals	*Metazoa*	106,843	102,155	98,070	2	35,213
Human	*Homo sapiens*	20,421	20,421	20,358	2	34,350
Plants	*Viridiplantae*	39,930	38,014	35,093	5	5,400
Mouse-ear cress	*Arabidopsis thaliana*	15,856	15,829	15,768	5	5,400
Fungi	*Fungi*	34,084	33,841	32,431	3	11,842
Yeast	*Saccharomyces cerevisiae*	7,919	7,912	7,290	16	4,910
Prokaryotes	*Bacteria*	334,009	332,477	255,373	7	10,746
Bacteria gram-	*Escherichia coli*	23,138	23,121	10,153	7	3,289
Bacteria gram+	*Staphylococcus aureus*	10,175	10,164	3,171	9	10,746
Archaea	*Archaea*	19,554	19,482	18,452	25	9,159
Viruses	*Viruses*	16,858	16,400	15,607	11	7,182

**Figure 3:**
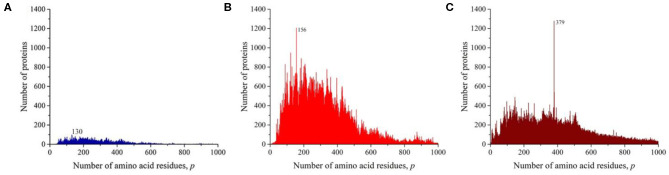
The distribution of the number of unique sequences on the Swiss-Prot database in various biological domains. **(A)** Archaea. **(B)** Prokaryotes. **(C)** Eukaryotes. Colors in Figures 3–8: **blue**, archaea; **red**, prokaryotes; **brown**, all eukaryotes and animals; **violet**, fungi; **green**, plants; **orange**, viruses.

We sequentially isolated and analyzed unique sequences of taxonomic groups of various levels from the entire mass of sequences from the Swiss-Prot database. At the first and highest level, the domains of archaea, prokaryotes, and eukaryotes were chosen (Woese et al., 1990). Despite the fact that the number of sequences in these domains varies significantly (Table 1), the general distribution pattern (Figure 3) is the same for them. Moreover, the overwhelming majority of amino acid residue sequences, as before, are concentrated in the range 2 ≤ *p* ≤ 1,000 (99.1% in archaea, 98.7% in prokaryotes, and 92.7% in eukaryotes). In archaea (Figure 3A), there is only one small visible peak at *p* = 130; in prokaryotes (Figure 3B), there are quite a lot of these peaks, and the peak is especially distinguished at *p* = 156 represent a large set of very different proteins; and in eukaryotes (Figure 3C), most of the peaks are not too large, except for one at *p* = 379.

**Figure 4:**
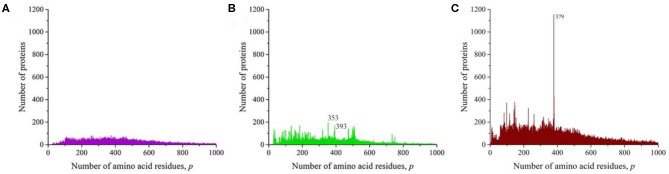
The distribution of the number of unique sequences on the Swiss-Prot database in various eukaryotes. **(A)** Fungi, **(B)** Plants, **(C)** Animals.

At the next taxonomic level, the unique amino acid residue sequences of the kingdoms (fungi, plants, and animals) were separately analyzed (Figure 4). The general nature of the obtained distributions indicates that noticeable peaks are practically unobservable in fungi (Figure 4A), while in plants, there are quite a lot of them (Figure 4B), and in the case of animals, a number of peaks also stand out, among which the peak at *p* = 379 is brighter than in the case of the distribution for eukaryotes (Figure 3C).

**Figure 5:**
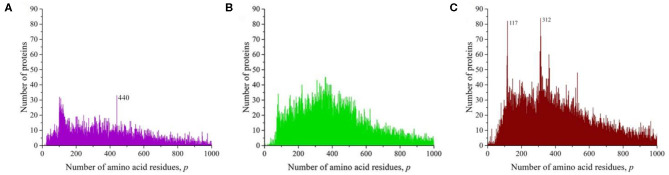
The distribution of the number of unique sequences on the Swiss-Prot database in individual representatives of eukaryotes (fungi, plants, animals). **(A)** *Saccharomyces cerevisiae*, **(B)** *Arabidopsis thaliana*, **(C)** *Homo sapiens*.

We also analyzed the unique amino acid residue sequences of individual representatives of fungi, plants, and animals (Figure 5). In the case of yeast (*Saccharomyces cerevisiae*), a distribution was obtained with a noticeable peak at *p* = 440 (Figure 5A). It turned out that most of the sequences with this number of amino acid residues are various proteins known as transposon polyproteins. Several not very large peaks are manifested in mouse-ear cress (Figure 5B). At the same time, two peaks clearly stand out in human (Figure 5C). One of them at *p* = 117 is more than 50% composed of various immunoglobulins; in the case of the other at *p* = 312, also more than half characterizes the presence of a large number of olfactory receptor proteins.

**Figure 6:**
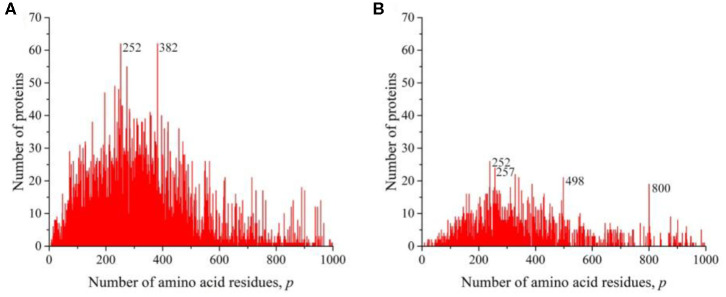
The distribution of the number of unique sequences on the Swiss-Prot database in bacteria. **(A)** Gram-negative *Escherichia coli*, **(B)** Gram-positive *Staphylococcus aureus*.

**Figure 7:**
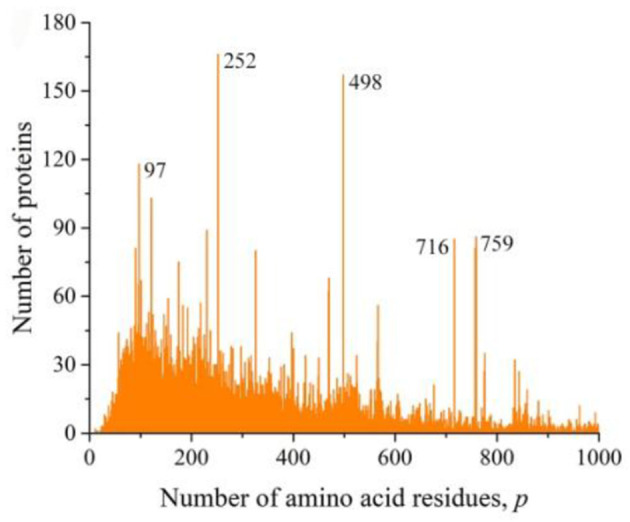
The distribution of the number of unique sequences in viruses on the Swiss-Prot database.

We also obtained distributions for a combination of sequences of Gram-negative (Figure 6A) and Gram-positive (Figure 6B) bacteria and for all viruses (Figure 7). All these distributions are characterized by a large number of noticeable peaks. An examination of primary structures corresponding to bacterial protein peaks represents a large set of very different proteins except two peaks in the distribution of viral sequences. The peak at *p* = 252 is particularly prominent (see Figures 1A,B), in which a significant part is occupied by the matrix protein of influenza A virus, which plays a key role in the replication of viruses, and the peak at *p* = 498 almost completely corresponds to the nucleoprotein protein (also in influenza A virus), which protects viral RNA from nucleases.

## Common Consideration of Protein Length Distribution

Obviously, the nature of any distribution needs explanation, which could best be made on the basis of an analytical mathematical expression derived from general considerations about the nature of peptide sequences. Obtaining such an expression is still faced with great difficulties, since many factors must be taken into account: details of the genesis of proteins, features of its constituent elements (physicochemical diversity of amino acid residues), evolutionary factors, and much more (Zamyatnin, 2003, 2004).

However, the fitting mathematical expression, which allows one to obtain curves that are as close as possible to real distributions, has been carried out repeatedly. Thus, for a number of individual organisms, the log-normal distribution function was used, which satisfactorily described the data for 13 species of bacteria, 4 archaea, and 1 eukaryote (Ramakumar, 1999).

The same approach was applied to describe the protein distributions of 1,302 species of prokaryotes and 140 eukaryotes (Tiessen et al., 2012). In this study, in addition to the log-normal distribution, a gamma-type distribution was also employed. The combined use of the log-normal and gamma distributions (Jhang, 2000) allowed the authors to conclude that the average length of eukaryotic proteins is greater than that of prokaryotes. A similar conclusion is illustrated in our present work when considering Figures 3B,C. The linguistic model of Menserath–Altmann was also used no less successfully (Menzerath, 1954; Altmann, 1980). The data for 10 proteomes with help from this model were described (Eroglu, 2014).

## Unique Length Distribution for One Protein Type

**Figure 8:**
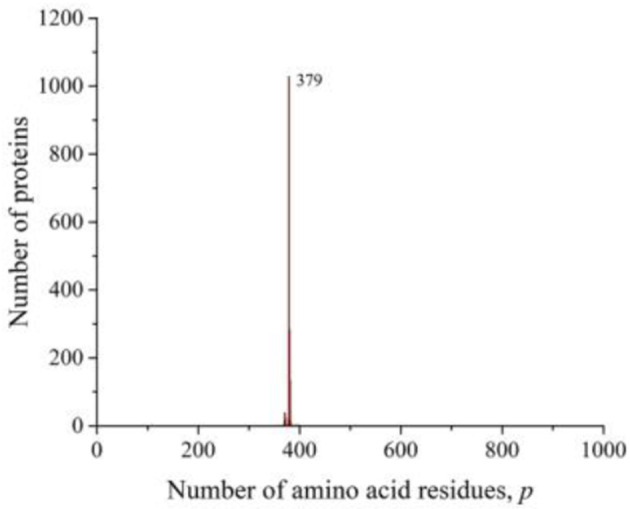
The distribution of the number of unique sequences of cytochrome *b* in eukaryotes in the Swiss-Prot database.

However, in all of these works, the main attention was paid to smoothing the distribution curves in order to fit certain mathematical models. At the same time, it follows from our results that numerous peaks in almost all taxonomic groups and individual species of living organisms carry additional information about the sequences that form these distributions. A striking example is the peak traced in the distribution of all amino acid residue sequences on the Swiss-Prot database (Figure 2C), eukaryotes (Figure 3C), and animals (Figure 4C). As already noted, this peak is a collection of mitochondrial cytochromes *b* having the same length of 379 amino acid residues and found only in animals. An additional analysis of the length distribution of animal cytochromes *b* (Figure 8) showed that these proteins have a predominant length of 379 amino acid residues (1,028 proteins). With a slightly smaller number of residues, there are 99 of these proteins and with a large number of 440.

For a large number of differing amino acid residue sequences, but with the same *p*-value, cytochrome *b* is precisely typical as being representative of animals. Swiss-Prot contains data on nearly 1,700 different sequences of this protein, obtained not only from animals. They are present in bacteria, plants, and fungi. However, all known non-animal cytochromes *b* always contain more than 379 amino acid residues. It can be assumed that the minimum value of this transmembrane protein in the evolutionary process could be achieved as a result of selection, which led to the most optimal sizes of all its eight sites penetrating the membrane (Esposti et al., 1993). Such examples may indicate that the most perfect protein lengths were selected in the evolutionary process to perform this function. As a result, many molecules with different sequences of the same length and characterized by the same functions were formed.

Obviously, the diversity of the functional properties of proteins is based on the diversity of the primary structures of their molecules. Many examples can be found where small peptide molecules of the same length (oligopeptides) and with the same *p* and different sequences have the same functions (Zamyatnin, 1991b,c, 2018b), e.g., pentapeptides met-enkephalin YGGFM and leu-enkephalin YGGFL) are the natural ligand for opiate receptor (Hughes et al., 1975). In this work, we particularly demonstrated that there are many different primary structures of polypeptides (proteins) of the same length, with the same functions.

## Future Directions

The UniProt and Swiss-Prot databases are constantly updated. However, there is already a large amount of information contained on them, which allows for many new and various analyses of the relationship between size, amino acid residue sequences, and many other physicochemical characteristics of natural sequences with the numerous functional properties of these molecules.

## Data Availability Statement

The datasets generated for this study are available on request to the corresponding author.

## Author Contributions

AZ performed all the experiments, treatment of their results, and wrote the manuscript. TB contributed to the biological consideration of the results and in writing the article. All authors contributed to the article and approved the submitted version.

## Conflict of Interest

The authors declare that the research was conducted in the absence of any commercial or financial relationships that could be construed as a potential conflict of interest.
